# DO RECOMMENDATIONS FROM REHABILITATION THERAPISTS MOTIVATE OLDER ADULTS TO INSTALL HOME MODIFICATIONS?

**DOI:** 10.1093/geroni/igae098.0009

**Published:** 2024-12-31

**Authors:** Mengzhao Yan, Bernard A Steinman, Jon Pynoos

**Affiliations:** University of Southern California, Los Angeles, California, United States; University of Wyoming, Laramie, Wyoming, United States; University of Southern California, Los Angeles, California, United States

## Abstract

With the rapid growth of the aging population, private homes have increasingly become an alternative setting for the provision of long-term care (LTC). For many, LTC needs increase during recovery from acute medical events and subsequent rehabilitation services. An estimated 20% of older Americans undergo physical rehabilitation services, including physical therapy, occupational therapy, and speech therapy, that can help to promote function and improve their ability to carry out daily activities. Often, rehabilitation therapists may recommend that older adults receiving their services make home modifications (HMs) to facilitate their recovery and improve home accessibility and safety. Nevertheless, it is unclear to what extent receiving recommendations is associated with decisions by older adults to install HMs. Guided by Andersen’s healthcare utilization model, which assesses factors that lead to the use of health services, we hypothesized that those who received recommendations from rehabilitation therapists would be more likely to modify their homes to accommodate disability. We analyzed data from the most recent round (2022) of the National Health and Aging Trends Study (NHATS). We used multivariate logistic regression to estimate the relationship between the recommendation and implementation of shower/tub and toilet HMs. Results indicated that older adults who received recommendations to modify their showers/tubs had 158% higher odds of installing shower/tub HMs, whereas those who received recommendations to modify areas around their toilets had 160% higher odds of installing toilet HMs. Findings suggest that older adults may be especially motivated to implement HM regimens when directed by rehabilitation professionals.

